# Outcomes for 298 breastfed neonates whose mothers received ketamine and diazepam for postpartum tubal ligation in a resource-limited setting

**DOI:** 10.1186/s12884-021-03610-1

**Published:** 2021-02-09

**Authors:** Mary Ellen Gilder, Nay Win Tun, Annabelle Carter, Ferdinand Frederik Som Ling Tan, Aung Myat Min, Hsa Eh, Pan Aye, Verena I. Carrara, Chaisiri Angkurawaranon, Rose McGready

**Affiliations:** 1 Department of Family Medicine, Faculty of Medicine, Chiang Mai University, Chiang Mai, Thailand; 2 Shoklo Malaria Research Unit, Mahidol-Oxford Tropical Medicine Research Unit, Faculty of Tropical Medicine, Mahidol University, Mae Sot, Tak Thailand; 3 Hospital Nij Smellinghe, Compagnonsplein 1, 9202 NN Drachten, Netherlands; 4 Centre for Tropical Medicine and Global Health, Nuffield Department of Medicine, University of Oxford, Oxford, UK

**Keywords:** Ketamine, Diazepam, Lactation, Safety, Resource-limited, Tubal ligation, Anesthesia

## Abstract

**Background:**

Anesthesia in lactating women is frequently indicated for time-sensitive procedures such as postpartum tubal ligation. Ketamine and diazepam are two of the most commonly used anesthetic agents in low resource settings, but their safety profile in lactating women has not been established.

**Methods:**

Medical records of post-partum tubal ligations between 2013 and 2018 at clinics of the Shoklo Malaria Research Unit were reviewed for completeness of key outcome variables. Logistic regression identified presence or absence of associations between drug doses and adverse neonatal outcomes: clinically significant weight loss (≥95th percentile) and neonatal hyperbilirubinemia requiring phototherapy.

**Results:**

Of 358 records reviewed, 298 were lactating women with singleton, term neonates. There were no severe outcomes in mothers or neonates. On the first postoperative day 98.0% (290/296) of neonates were reported to be breastfeeding well and 6.4% (19/298) had clinically significant weight loss. Phototherapy was required for 13.8% (41/298) of neonates. There was no association between either of the outcomes and increasing ketamine doses (up to 3.8 mg/kg), preoperative oral diazepam (5 mg), or increasing lidocaine doses (up to 200 mg). Preoperative oral diazepam resulted in lower doses of intraoperative anesthetics. Doses of intravenous diazepam above 0.1 mg/kg were associated with increased risk (adjusted odds ratio per 0.1 mg/kg increase, 95%CI) of weight loss (1.95, 95%CI 1.13–3.35, *p* = 0.016) and jaundice requiring phototherapy (1.87, 95%CI 1.11–3.13, *p* = 0.017).

**Conclusions:**

In resource-limited settings ketamine use appears safe in lactating women and uninterrupted breastfeeding should be encouraged and supported. Preoperative oral diazepam may help reduce intraoperative anesthetic doses, but intravenous diazepam should be used with caution and avoided in high doses in lactating women.

## Introduction

Information about the safety of ketamine and diazepam in lactating women is limited, despite their designation as essential drugs by the WHO [1]. When the safety profile of anesthetic agents in lactation is unknown, both over- and under-caution confer risk to the mother-infant dyad. Over-cautious guidelines may unnecessarily withhold breastmilk, anesthesia, or operative procedures in the setting of lactation, while under-cautious guidelines may expose infants to dangerous levels of drugs via breast milk. Due to the short half-life, minimal presence in breast milk, and limited oral bioavailability of many anesthetic agents, once a mother is awake and able to breastfeed her baby, breastfeeding after anesthesia is generally considered safe [2–5].

Infant feeding and family planning are two of the most important issues for a postpartum woman. Breastfeeding confers a host of benefits to mother and child, especially in resource-limited settings [6], where breast milk is often the only safe source of neonatal nutrition. Prevention of unintended pregnancies can also save both mother and child lives [7]. Postpartum tubal ligation under anesthesia is a safe and highly effective method of contraception for a woman whose family is complete, and contraceptive procedures are increasingly recognized as essential services [8].

Despite over 50 years of clinical use [9] and wide availability in low- and middle-income countries, including for caesarean section, there is no data in the scientific literature on the safety of ketamine in lactation [2–5]. Ketamine’s sympathomimetic effects prevent the hypotension and apnea associated with other anesthetic agents, allowing for its use in settings without capacity for mechanical ventilation or administration of vasopressors [10]. It has a wide therapeutic range and is safely used as an anesthetic for patients in all age groups and for a variety surgical procedures [11, 12]. In addition, recent research on ketamine as prophylaxis for postpartum depression [13, 14] has created interest in the question of ketamine excretion in breastmilk, and a pharmacodynamic study in breastfeeding women is underway (ClinicalTrials.gov identifier: NCT04285684).

Breastmilk drug concentrations can only be determined conclusively by pharmacodynamic data. Ketamine’s moderate lipophilicity, molecular weight and relatively low protein binding all suggest that it may be present in the breast milk. However, as it is cleared rapidly from the body and has low oral bioavailability, excreted amounts in breast milk are unlikely to accumulate or cause significant effects on the breastfeeding infant. Reassuringly, it has been used safely as an analgesic and anesthetic for preterm neonates [15].

Ketamine side effects include emergence phenomena such as hallucinations, agitation, and panic attacks. To minimize these unwanted effects, it is often given with other anesthetic agents such as diazepam [16].

Diazepam is widely available due to its use in emergency treatment of status epilepticus [17], and is an anxiolytic and an adjuvant anesthetic agent. Several small studies with one to four women receiving repeated maternal doses showed concentrations in breast milk (including colostrum) ranging from 15 to 200 mcg/L [8–11]. Higher milk concentrations were found with higher maternal doses and chronic use, with relative infant doses (RID) of up to 9% of weight adjusted maternal doses. In contrast, a single dose of 2.5–10 mg diazepam IV given during sterilization procedures 1 month postpartum resulted in undetectable amounts of diazepam and metabolites in breastmilk and infant blood (lower limit of detection 150 mcg/L) in 8 subjects. This is consistent with a maximum RID of 3% of the weight adjusted maternal dose [18]. Based on this, single diazepam doses given intraoperatively have been thought likely to be safe [19], though controversy remains [20].

Effects of diazepam include sedation and respiratory depression at high doses. Importantly, diazepam displaces protein-bound bilirubin, potentially leading to clinical jaundice in neonates [21]. Concerns about sedation [22] and hyperbilirubinemia [23] in breastfeeding infants of women receiving repeated doses of diazepam have been raised.

On the Thailand-Myanmar border initiation of breast feeding is high [24] as are the rates of neonatal hyperbilirubinemia (NH) which affected 16.7% of term (EGA ≥ 37 weeks) neonates of multiparous mothers in a recent prospective cohort [25]. The acceptability and cost-effectiveness of locally available tubal ligation in this area has been published previously [26]. Ketamine and diazepam were the only available general anesthetic agents, and maintenance of supply of these essential medications was challenging.

Here we present a retrospective analysis of 298 infants of mothers who received ketamine, diazepam and lidocaine while undergoing tubal ligation for sterilization. Only singleton, term neonates who were breast fed were included for analysis of outcomes including: clinically significant weight loss (CSWL), NH, and, where available, neurodevelopment at 1 year.

## Methods

### Study design

The aim of this study was to describe the clinical outcomes of neonates who were potentially exposed to ketamine, diazepam and lidocaine in maternal breast milk, and identify any relationships between anesthetic doses and commonly observed adverse neonatal outcomes. Clinical data extracted from the records of women undergoing postpartum tubal ligation and their infants was explored and analyzed using logistic regression to compare infant outcomes against maternal anesthetic drug doses.

### Setting

Records of tubal ligations performed at clinics of the Shoklo Malaria Research Unit (SMRU) between 2013 and 2018 were reviewed. The clinics are located in Tak Province, Thailand, and provide free antenatal, delivery, family planning, and general medical care to rural migrant workers from Myanmar. Contraceptive options include combined oral contraceptives, medroxyprogesterone acetate injection, contraceptive implant, copper intrauterine device and postpartum tubal ligation.

Tubal ligations are performed by qualified medical doctors with appropriate surgical experience. Locally trained skilled birth attendants (training reported elsewhere [27]) support as surgical assistants, take vital signs, and administer medications at the doctor’s request.

There have not been any specific guidelines about breastfeeding after anesthesia at SMRU clinics. Women were encouraged to breastfeed when they were awake enough to do so, usually within 2–4 h following the procedure. During the operation, small amounts of milk were provided (e.g. 10–30 ml) by syringe if babies were crying. Syringe feeding of larger amounts was used based on clinical indications (weight loss or severe jaundice), and was usually maternal breast milk but formula was allowed when maternal breast milk was insufficient.

Available anesthetics were lidocaine, diazepam and ketamine, and most operations were performed with a combination of these three medications. A loading dose of 1 mg/kg ketamine was used for induction, followed by 0.5 mg/kg as needed (usually to 1.5–2.5 mg/kg total dose). Diazepam was available as 5 mg tablets and 10 mg (2 ml) vials, and was usually given as 5 mg per dose. For crude analysis doses were divided into low and high dose categories. Low dose ketamine was defined as < 1.16 mg/kg and high dose was ≥1.16 mg/kg maternal body weight, based on a median dose of 1.16 mg/kg in this cohort. A threshold of 1 mg/10 kg, approximately a 5 mg IV dose, was used for diazepam: low dose IV diazepam was defined as < 1 mg/10 kg, and high dose was ≥1 mg/10 kg maternal body weight.

CSWL was defined as reaching or exceeding the 95th percentile of weight loss for the respective day of life compared to a historical cohort of infants of multiparous women in a similar population [28], the day after the operation. Infants were weighed each morning on Seca scales with 5 g precision.

NH in this report is defined as hyperbilirubinemia requiring phototherapy. From 2008 onwards phototherapy units [29], total serum bilirubin (SBR) measurement and G6PD assessment were available for jaundiced newborns. G6PD deficiency is a major cause of NH in this population and was assessed using the fluorescent spot test. An on-site bilirubinometer measured capillary blood SBR and phototherapy was started when SBR results exceeded thresholds based on the UK NICE guidelines [30]. A prospective study of jaundice at SMRU [31] from 2015 to 16 resulted in screening of SBR and G6PD for all neonates before discharge.

Staff assessed low risk infants daily and high-risk infants 6 hourly; the soonest evaluation after breastfeeding resumed following anesthesia was taken. Parity was defined as number of infants born at an estimated gestational age (EGA) of 28 weeks or more. EGA at delivery was estimated by ultrasound before 24 weeks of gestation, and by Dubowitz exam if an early ultrasound was not available. Apgar scores were calculated by skilled birth attendants at delivery. Blood loss was measured by weight and postpartum hemorrhage was defined as blood loss of more than 1 l. SGA was calculated using Intergrowth-21 standards [32]. Body mass index (BMI) categories followed those recommended for use in Asian populations [33].

Neurodevelopmental data from prospective infant cohort studies [31, 34] was included when available. Inclusion in these studies was not related to contraceptive choice or any of the variables in this study. Neurodevelopment at 1 year was assessed using the locally developed and validated Shoklo Developmental Test [35, 36].

### Data extraction

Operation logbooks and notes were both searched to identify eligible women. Women were included if they had term, live, singleton infants who were breastfeeding on the day of the operation and for whom data on the variables of interest was available. Extracted data was entered into a STATA 15 (StataCorp, College Station, TX, USA) data file.

### Statistical analysis

Data was analyzed using STATA 15. Chi-squared test was used for comparison of binary variables with binary outcomes. Non-parametric tests of association were used for crude comparisons of non-normally distributed variables: Mann-Whitney *U* for binary outcomes and Spearman’s correlation coefficient for continuous outcomes.

A causal model was drawn and crude associations with weight loss and NH were investigated before creating the logistic regression models. Logistic regression was used for relationships between drug doses and the binary outcomes (weight loss and NH) and linear regression was used for continuous neurodevelopmental scores. Theoretical and statistical factors were considered and any variable whose inclusion in the model changed the effect estimate by 10% or more was automatically included.

Systemic anesthetic agents (ketamine and both intravenous and oral diazepam) were included in both the weight loss and NH regression models a priori*.* Lidocaine was excluded from the model due to lack of impact on effect estimates, and missing values (lidocaine dose was not always recorded). For the weight loss model, postpartum hemorrhage, Apgar scores, and EGA were included as a priori confounders*.* For the NH model maternal factors of Karen ethnicity, pre-eclampsia and obesity, and infant factors of sex and EGA were considered a priori confounders [31, 37].

## Results

**Fig. 1:**
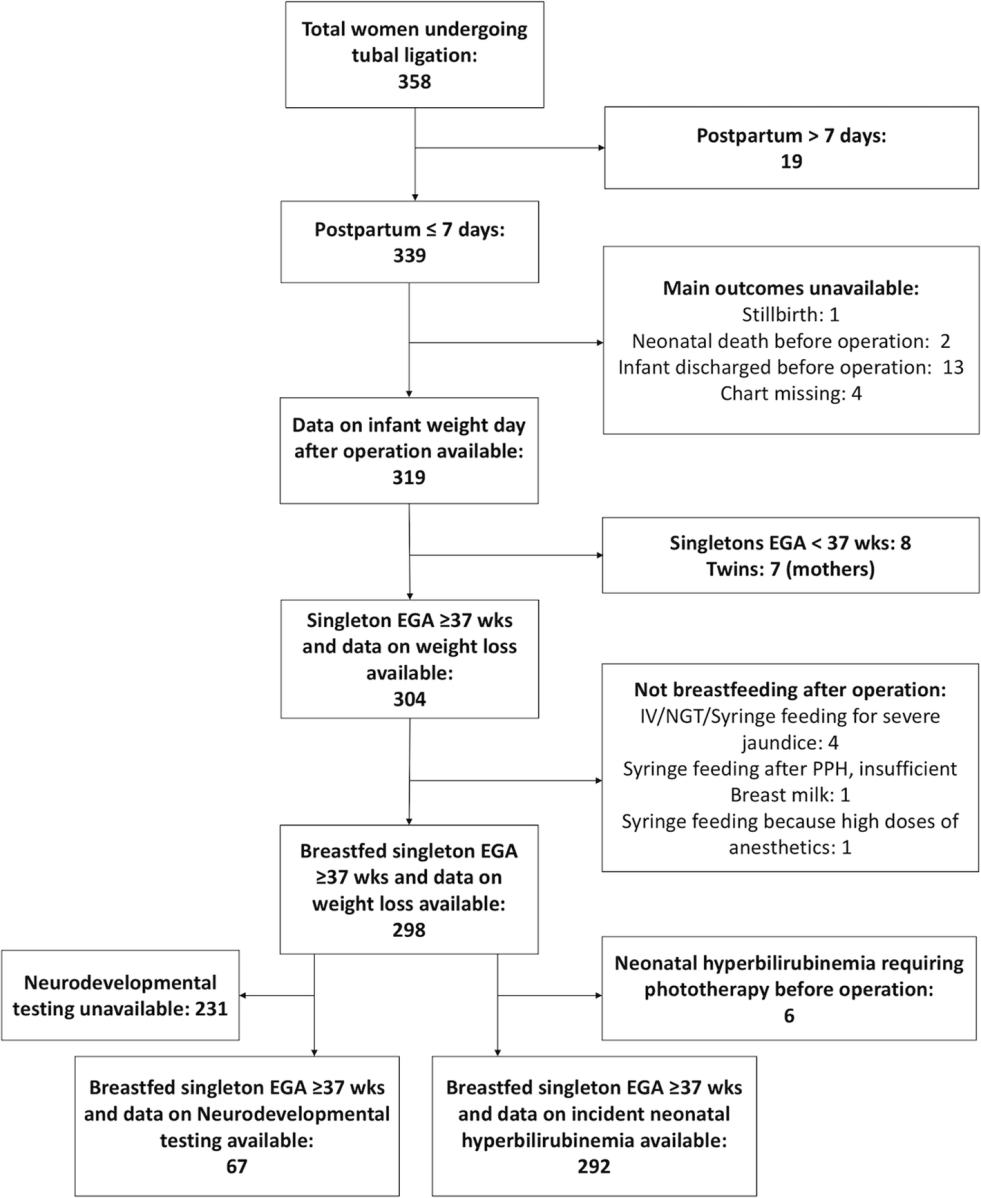
Study Inclusion Flow

Over 350 records were reviewed (Fig. 1) and 298 eligible term singletons identified. Median parity of women receiving tubal ligation was 5 and infant condition at birth was generally good (Table 1). No seizures or neonatal deaths occurred following exposure to maternal anesthesia.

**Table 1:** Demographics and anesthetic drug doses

Variable	n	
Maternal Age, (years)^b^	298	33 (21–45), [30–37]
Maternal Parity^b^	298	5 (1–12), [4–6]
EGA at delivery (weeks+days)^b^	298	39 + 5 (37 + 0 to 43 + 3), [39 + 0 to 40 + 2]
Birth weight (grams)^b^	298	3123 (2020–5040), [2860–3400]
5 min Apgar score^b^	296	10 (7–10), [10-10]
Postpartum day of operation^a, b^	298	2 (1–6), [2, 3]
Maternal weight on day of operation (kg)^b^	294	52 (33–80), [47–59]
Received IV ketamine^c^	298	297/298 (99.7)
IV ketamine dose (mg/kg maternal body wt)^b^	292	1.16 (0.46–3.80), [1.00–1.53]
Received IV diazepam^c^	298	246/298 (82.6)
IV diazepam dose (mg/10 kg maternal body wt)^b^	241	1.70 (0.29–6.15), [1.14–2.00]
Received PO diazepam^c^	298	148/298 (49.7)
Oral diazepam dose (mg)^b^	147	5 (5–10), [5-5]
Lidocaine, local to incision, dose (mg)^b^	290	100 (50–200), [100–100]

Note: weight was missing for 5 patients. Doses were missing for one patient who was documented to have received PO and IV diazepam and IV ketamine

*Abbreviations*: *EGA* estimated gestational age, *IV* intravenous, *PO* oral, *mg* milligrams, *kg* kilograms, *wt* weight

^a^counted as calendar days with the date of birth considered “day 1”

^b^Median (range), [interquartile range]

^c^n (%)

### Anesthetic practice

Median (range) ketamine dose was 1.16 mg/kg (0.46–3.80), and IV diazepam was 1.70 mg/10 kg (0.29–6.15) (Table 1). There was a strong positive correlation between IV diazepam doses and IV ketamine doses, reflecting higher doses of both in longer procedures, or when adequate anesthesia effect was difficult to achieve. Over the study period, gradually lower doses of IV diazepam and higher doses of ketamine were used. When oral diazepam was given preoperatively (15-60 minutes before the planned operation start time) lower doses of intraoperative anesthetics were used. Median [IQR] ketamine doses in cases where PO diazepam was given was 1.11 [0.98–1.5] compared with 1.26 [1.00–1.60] mg/kg (*p* = 0.022) in cases without PO diazepam. Likewise, IV diazepam in cases where PO diazepam was given was 1.33 [0–1.85] vs 1.75 [0.98–2.04] mg/10 kg (*p* = 0.004) in cases without PO diazepam. Lidocaine 100 mg injected into the skin and soft tissue was standard, and use of higher doses did not result in lower doses of systemic anesthetic agents.

### Breastfeeding practices

The majority of infants were fully breastfed. Six infants were excluded from analysis because feeding on the day of the operation was done by syringe, nasogastric tube or intravenous fluids because of: severe jaundice (4 infants), insufficient maternal breastmilk (1 infant) and doctor concern about high doses of anesthetic (1 infant).

### Infant outcomes

**Fig. 2:**
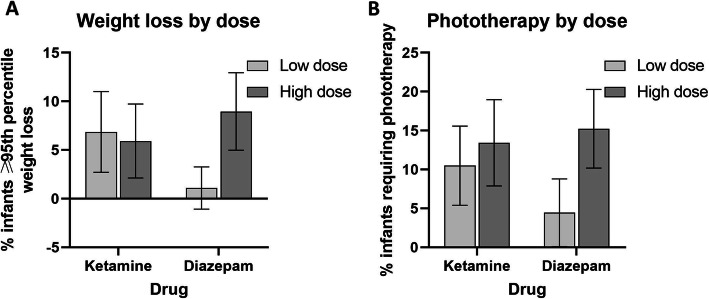
**a** Crude relationship between clinically significant weight loss (≥95th percentile) and low vs high doses of ketamine and diazepam. **b** Crude relationship between neonatal hyperbilirubinemia requiring phototherapy and low vs. high doses of ketamine and diazepam. Low dose ketamine (*n* = 146) was defined as < 1.16 mg/kg and high dose (*n* = 147) was ≥1.16 mg/kg maternal body weight. Low dose IV diazepam (*n* = 92) was defined as < 1 mg/10 kg maternal body weight, and high dose (*n* = 201) was ≥1 mg/10 kg maternal body weight. **a** Chi squared test gave *p* = 0.801 for comparison of low vs high dose ketamine and *p* = 0.011 for comparison of low vs high dose diazepam. **b** Chi squared test gave *p* = 0.478 for ketamine and *p* = 0.009 for diazepam

Overall, 6.4% (95%CI 3.6–9.2%, 19/298), infants had CSWL on the day after the operation (Table 2) and 13.8% (95%CI 9.8–17.7%, 41/298) required phototherapy. After excluding six infants with NH before the operation, there was incident NH in 11.9% (35/292) infants following exposure (Table 3). On crude analysis, there was no difference in either neonatal outcome at higher doses of ketamine (≥1.16 mg/kg maternal body weight) compared with lower doses. High dose (≥1 mg/10 kg maternal body weight) IV diazepam was associated with an almost 9-fold increased risk of clinically significant weight loss, and more than a 3-fold increased risk of NH (Fig. 2) on univariate analysis.

**Table 2:** Univariate analysis of factors associated with clinically significant weight loss (CSWL) ≥ 95th percentile

**Continuous Variables**	**No CSWL**	**CSWL**	**Crude OR (95% CI)**	***p*** **value**
Ketamine dose (mg/kg)^a^ *N* = 293	1.16 [0.46–3.80]	1.14 [0–2.27]	0.92 (0.34–2.49)	0.869
IV diazepam dose (mg/10 kg)^a^ *N* = 293	1.47 [0–4.35]	1.82 [0.96–6.15]	1.94 (1.20–3.16)	**0.007**
Lidocaine dose (mg)^a^ *N* = 290	100 [50–200]	100 [100–150]	1.00 (0.98–1.02)	0.801
**Categorical Variables**		**Percent (n) CSWL**	**Crude OR (95% CI)**	***p*** **value**
PO diazepam *N* = 297	No	8.0 (12/150)	*Reference group*	
	Yes	4.8 (7/147)	0.58 (0.22–1.50)	0.259
Maternal age (years) *N* = 298	≤33	6.6 (10/152)	*Reference group*	
	> 33	6.2 (9/146)	0.93 (0.37–2.37)	0.884
Parity *N* = 298	≤ 2	7.5 (5/67)	*Reference group*	
	> 2	6.1 (14/231)	0.80 (0.28–2.31)	0.680
BMI *N* = 298	≥ 18.5	5.2 (14/271)	*Reference group*	
	< 18.5	18.5 (5/27)	4.17 (1.37–12.66)	**0.012**
Postpartum hemorrhage > 1 L *N* = 298	No	6.2 (18/291)	*Reference group*	
	Yes	14.3 (1/7)	2.53 (0.29–22.14)	0.402
5 Minute Apgar Score *N* = 296	9 or 10	6.1 (18/294)	*Reference group*	
	≤8	50 (1/2)	15.33	0.057
Infant sex *N* = 298	Male	5.5 (9/163)	*Reference group*	
	Female	7.4 (10/135)	1.37 (0.54–3.47)	0.509
SGA *N* = 298	No	6.0 (15/249)	*Reference group*	
	Yes	8.2 (4/49)	1.39 (0.44–4.37)	0.577
EGA at birth *N* = 298	≥ 39 wks	6.2 (14/226)	*Reference group*	
	37- < 39 wks	6.9 (5/72)	1.13 (0.39–3.25)	0.821
Year of birth *N* = 298	2013–2015	6.7 (12/180)	*Reference group*	
	2016–2018	5.9 (7/118)	0.88 (0.34–2.31)	0.800
Complicated/ difficult operative procedure *N* = 298	No	5.9 (16/273)	*Reference group*	
	Yes	12.0 (3/25)	2.19 (0.59–8.10)	0.240
Postpartum day of tubal ligation *N* = 298	0–2	5.6 (12/216)	*Reference group*	
	3–7	8.5 (7/82)	1.59 (0.60–4.18)	0.350

*Abbreviations*: *CSWL* clinically significant weight loss, *EGA* estimated gestational age, *IV* intravenous, *PO* per oral

^a^median [range]

**Table 3:** Univariate analysis of factors associated with neonatal hyperbilirubinemia (NH) requiring phototherapy

**Continuous Variables**	**No NH**	**NH**	**Crude OR (95% CI)**	***p*** **value**
Ketamine dose (mg/kg)^a^ *N* = 287	1.33 [0.46–3.80]	1.40 [0–3.07]	1.32 (0.65–2.68)	0.429
IV diazepam dose (mg/10 kg)^a^ *N* = 287	1.34 [0–4.35]	1.85 [0–6.15]	1.83 (1.22–2.74)	**0.003**
Lidocaine dose (mg)^a^ *N* = 285	105.86 [50–200]	108.29 [90–200]	1.00 (0.99–1.02)	0.571
**Categorical Variables**		**Percent (n) NH**	**Crude OR (95% CI)**	***p*** **value**
PO diazepam *N* = 291	No	13.0 (19/146)	*Reference group*	
	Yes	11.0 (16/145)	0.83 (0.41–1.68)	0.604
Karen Ethnicity *N* = 292	No	12.8 (20/156)	*Reference group*	
	Yes	11.0 (15/136)	0.84 (0.41–1.72)	0.639
Pre-eclampsia *N* = 292	No	11.4 (30/263)	*Reference group*	
	Yes	17.2 (5/29)	1.62 (0.57–4.56)	0.363
Maternal obesity *N* = 292	No	10.4 (27/260)	*Reference group*	
	Yes	25.0 (8/32)	2.88 (1.18–7.03)	**0.021**
Infant sex *N* = 292	Male	11.3 (18/159)	*Reference group*	
	Female	12.8 (17/133)	1.15 (0.57–2.33)	0.702
EGA at birth *N* = 292	≥ 39 wks	9.5 (21/222)	*Reference group*	
	37- < 39 wks	20.0 (14/70)	2.39 (1.14–5.01)	**0.021**
Weight loss ≥95^th^percentile 1st post-operative day *N* = 292	No	11.7 (32/274)	*Reference group*	
	Yes	16.7 (3/18)	1.51 (0.42–5.51)	0.531
Year of birth *N* = 292	2013–2015	13.2 (23/174)	*Reference group*	
	2016–2018	10.2 (12/118)	0.74 (0.35–1.56)	0.432
Postpartum day of tubal ligation *N* = 292	0–2	12.1 (26/214)	*Reference group*	
	3–7	11.5 (9/78)	0.94 (0.42–2.11)	0.887
G6PD deficiency *N* = 100	No	26.7 (23/86)	*Reference group*	
	Yes	64.3 (9/14)	4.93 (1.50–16.25)	**0.009**

*Abbreviations*: *NH* neonatal hyperbilirubinemia requiring phototherapy, *EGA* estimated gestational age, *G6PD* glucose-6-phosphate deficiency, *IV* intravenous, *PO* per oral

^a^median [range]

Breastfeeding was assessed in 99.3% (296/298) of infants on the first day after the operation, and was described as “well”, or “strong” in 98.0% (290/296). General condition, tone or alertness was noted in 84.9% (253/298) of infants, and was “well” or “normal” in 98.0% (248/253). There was no difference in clinical assessments or vital signs between infants exposed to high vs. low doses of maternal anesthetic agents.

### Risk of excessive weight loss

There was no association between CSWL and ketamine dose up to a maximum dose of 3.8 mg/kg in this cohort. Higher IV diazepam dose was significantly associated with weight loss, almost doubling the odds ratio of excessive weight loss for each additional 1 mg diazepam per 10 kg maternal body weight on univariate analysis (Table 2). Increasing lidocaine doses, and preoperative oral diazepam were not associated with increased CSWL.

In this group of mostly mature multiparous women, neither maternal age nor parity appeared to have a significant impact on weight loss or the effect estimates for diazepam or ketamine (Tables 2 & 4). Low maternal body mass index (BMI) was associated with greater weight loss. There were very few infants assigned low Apgar scores in these high parity women, but there was a trend towards more weight loss in these infants with a 5 min Apgar score of 8 or below. Postpartum day of the operation, intraoperative complications, and year of the operation were not significantly associated with weight loss on univariate analysis. In the final multivariable analysis (Table 4), higher diazepam dose (AOR 1.95, 95% CI 1.13–3.35, per 1 mg/10 kg increase in dose) and low maternal BMI (AOR 3.97, 95% CI 1.18–13.39) were the only factors associated with increased risk of clinically significant weight loss.

**Table 4:** Multivariable analyses of factors associated with clinically significant weight loss and neonatal hyperbilirubinemia

Variable		Clinically significant weight loss ***N*** = 291		Neonatal hyperbilirubinemia ***N*** = 287	
		Adjusted OR (95% CI)	***p*** value	Adjusted OR (95% CI)	***p*** value
Ketamine dose (mg/kg)^a^		0.77 (0.31–1.92)	0.581	0.86 (0.38–1.94)	0.719
IV diazepam dose (mg/10 kg)^a^		1.95 (1.13–3.34)	**0.016**	1.87 (1.11–3.13)	**0.017**
PO diazepam	No	*Reference group*	–	*Reference group*	–
	Yes	0.70 (0.24–1.98)	0.497	1.11 (0.38–2.52)	0.803
Karen Ethnicity	No	–	–	*Reference group*	–
	Yes	–	–	0.80 (0.37–1.72)	0.561
Pre-eclampsia	No	–	–	*Reference group*	
	Yes	–	–	0.72 (0.19–2.65)	0.620
Maternal obesity (BMI ≥ 27.5 kg/m^2^)	No	–	–	*Reference group*	–
	Yes	–	–	3.71 (1.40–9.83)	**0.008**
Maternal Underweight (BMI < 18.5 kg/m^2^)	No	*Reference group*	**–**	–	–
	Yes	3.97 (1.18–13.37)	**0.026**	–	–
Postpartum hemorrhage > 1 L	No	*Reference group*	–	–	–
	Yes	4.38 (0.46–42.14)	0.200	–	–
5 Minute Apgar Score	9 or 10	*Reference group*	–	–	–
	≤8	6.53 (0.23–186.07)	0.272	–	–
Infant sex	Male	–	–	*Reference group*	–
	Female	–	–	1.11 (0.51–2.44)	0.782
EGA at birth	≥ 39 wks	*Reference group*	–	*Reference group*	–
	37- < 39 wks	0.75 (0.23–2.43)	0.634	2.15 (0.96–4.84)	0.064

*Abbreviations*: *EGA* estimated gestational age, *BMI* body mass index, *L* liter, *IV* intravenous, *PO* per oral

^a^median [range]

### Risk of neonatal hyperbilirubinemia requiring phototherapy (NH)

There were 292 infants eligible for analysis of incident NH, after exclusion of six infants who had NH before breastfeeding had resumed following the operation (Fig. 1). On univariate and multivariate analysis (Tables 3 & 4), there was no association between NH and increasing ketamine or lidocaine doses. Increasing IV diazepam dose, but not oral diazepam, was associated with increased risk for NH. On both univariate and multivariate analysis, there was a strong association between obesity and NH but no effect modification between IV diazepam dose and obesity. EGA but not sex was associated with NH in both univariate and multivariate analysis. Neither weight loss of ≥95th percentile nor ≥90th percentile on the day after the operation was associated with increased NH.

G6PD status was known for 100 infants, and the maternal weight was known for 98 of these. Regression included only primary exposures (ketamine and IV diazepam) and significant confounders (obesity, EGA and G6PD deficiency) in this small group. Controlling for G6PD deficiency did not change the lack of association between ketamine and NH (AOR 0.75, 95% CI 0.25–2.22, *p* = 0.598). The association between IV diazepam and NH remained (AOR 2.18, 95% CI 1.06–4.50, *p* = 0.035). As expected, G6PD deficiency conferred a considerable increased risk of NH in this group (AOR 3.96, 95%CI 1.00–15.65, *p* = 0.049).

### Neurodevelopmental outcomes

Neurodevelopmental testing results, done as part of concurrent clinical trials, were available for 66 infants in this cohort. There was no evidence of lower neurodevelopmental scores with increasing doses of maternal ketamine (OR 0.59, 95%CI − 0.87 to 2.04, *p* = 0.432) or IV diazepam (OR 0.50, 95%CI − 0.55 to 1.56, *p* = 0.346), and none of the scores fell below the threshold for concern. Median scores for the cohort were identical to those described in other datasets [35, 36].

## Discussion

This report is the first ever description of breastfed infants (*n* = 298) exposed to maternal ketamine, and describes the largest cohort of infants exposed to diazepam through maternal breast milk in the literature. The findings provide reassurance about the safety for breastfed infants of ketamine use in lactating women: the incidence of weight loss and jaundice in infants of women exposed to high and low doses of ketamine were no different, and comparable to the general population. Increasing doses of IV diazepam were associated with increased risk of weight loss and jaundice in breastfed infants, suggesting that caution should be used with higher doses of this medication. However, overall there were no severe events in this cohort of term infants.

The available information strongly suggests that current practice of using ketamine in breastfeeding women is safe for neonates, and ketamine should be preferred over diazepam if possible. There was no evidence of increased weight loss, jaundice, poor breastfeeding, sleepiness, seizures, mortality or long-term neurological damage in this cohort with doses up to 3.8 mg/kg. Ketamine’s short half-life likely contributes to the favorable outcomes: women receiving higher doses of ketamine are able to wake up and feed their infants sooner than those receiving higher doses of diazepam, and ketamine is rapidly cleared from their circulation leading to low breast milk concentrations. Since 1996, approximately 2000 lactating women have undergone tubal ligation [7] at SMRU clinics with ketamine anesthesia, and no signal of severe adverse events in breastfeed infants have been noted, adding confidence to these findings. The consistent evidence in this dataset of mild adverse effects of high doses of IV diazepam suggests that the study had adequate power to demonstrate any clinically significant effects of ketamine on breastfed infants if they did exist.

Higher doses of IV diazepam were associated with increased risk of CSWL and NH. Fortunately, there were no cases of kernicterus, seizures, neonatal mortality or long-term neurological damage in this cohort, and even among infants exposed to higher doses of diazepam NH did not exceed the expected incidence in the population. Diazepam’s long half-life likely contributed to mothers having more difficulty resuming breastfeeding post-operatively, contributing to weight loss. There was no evidence of infant sleepiness due to diazepam exposure, but this may have also contributed to weight loss. Almost one-third of women received no IV diazepam or low-dose (< 1 mg/10 kg) IV diazepam, suggesting that many operations can be successfully completed without resorting to high or repeated doses of diazepam.

Each increase of 1 mg/10 kg maternal body weight of IV diazepam almost doubled the risk of needing phototherapy. The increased incidence of NH unrelated to weight loss indicates that the babies who developed NH after the exposure received adequate breast milk following maternal surgery. Thus, the likely mechanism of NH was competitive displacement of protein-bound bilirubin by diazepam transferred to infant blood via breast milk rather than “breastfeeding jaundice”. Overall, at 13.8% (95% CI 10.2–18.3%) the incidence of NH in this cohort was lower but not significantly different to a recent prospective cohort of infants [31] in which 16.7% (95%CI 14.5–19.0, 179/1075) of infants of multiparous mothers required phototherapy.

This analysis supports the use of 5 mg oral diazepam preoperatively. This practice reduced intraoperative anesthetic doses and was not associated with adverse effects in the breastfed infants. Use of increased doses of local anesthetic up to 200 mg lidocaine (20 ml of 1% solution) was not associated with any adverse effects, but did not result in lower doses of IV anesthetics and therefore is not recommended. Some healthcare providers tell women to interrupt breastfeeding after lidocaine exposure, and this advice is potentially harmful and not supported by any evidence [38].

Recommendations:

- Ketamine anesthesia in lactating women does not endanger term breastfeeding neonates at doses up to 3.8 mg/kg. Breastfeeding should not be interrupted.
- Preoperative oral diazepam 5 mg may help reduce doses of intraoperative anesthetics and prevent emergence phenomena.
- Caution should be used with IV diazepam in lactating women. At doses > 1 mg/10 kg [0.1 mg/kg] monitoring for infant sedation and jaundice is recommended. Doses exceeding 0.1 mg/kg should not be used in lactating mothers of very vulnerable infants (e.g. preterm or with severe NH).
- Postoperative women are at risk for breastfeeding difficulties due to discomfort and drowsiness and should have extra support to ensure adequate frequency and duration of breastfeeding.

There are several limitations to this report from a resource-limited setting. No pharmacokinetic measurements of ketamine and diazepam in breastmilk were available to support the findings. There was no randomization of anesthetic drugs or doses, and many factors could affect surgeon decisions. However, involvement of multiple surgeons over the years captured significant variation in routine practice. There was no available control group for this cohort (healthy infants of mothers not undergoing tubal ligation were discharged at 24–48 h), but the historical weight loss cohort [28] provided a comparison group, and the variety of anesthetic doses allowed for comparisons to be made and dose-responses to be evaluated. Fifty-two women did not receive any IV diazepam, providing a small control group of infants not exposed to IV diazepam. Clinical assessments of infants were generally done 18–20 h after potential exposure, and transient effects on infant alertness and breastfeeding may have been missed. However, staff were continually present on the ward and excessive somnolence or poor feeding would have been noted. Though neurodevelopmental data at 1 year was limited to approximately one fourth of the total cohort, infants with testing were essentially a random sample as inclusion in clinical studies was not related in any way to drug dose or maternal choice for tubal ligation. Finally, this cohort only includes infants in the first week of life. Breast milk composition changes over time and the profile of ketamine in mature milk could differ from that in colostrum and transitional milk represented here. However, concerns about sedation, weight loss and jaundice are highest in the vulnerable neonatal period.

## Conclusions

Ketamine appears to be a safe anesthetic agent in postpartum lactating women at doses up to 3.8 mg/kg, but caution should be used with IV diazepam. Doses of intraoperative diazepam of more than 0.1 mg/kg were associated with CSWL and NH. Premedication with oral diazepam but not increasing doses of local anesthesia resulted in lower intraoperative anesthetic doses, and neither agent appeared to be harmful. The benefits of post-partum tubal ligation and maintenance of breastfeeding following ketamine and diazepam use outweighs the low risk of adverse neonatal effects from these anesthetic agents in low resource settings.

## Data Availability

The data presented in this article are available on request. Please contact Chaisiri Angkurawaranon (chaisiri.a@cmu.ac.th) or SMRU (Shoklo-unit.com) with a brief explanation of your research interest.

## Abbreviations

BMI: Body mass index
CSWL: Clinically significant weight loss
EGA: Estimated gestational age
G6PD: glucose-6-phosphate dehydrogenase
IV: Intravenous
kg: Kilograms
mg: Milligrams
ml: Milliliters
NH: Neonatal hyperbilirubinemia (requiring phototherapy)
PO: Per oral
RID: Relative infant dose (weight adjusted)
SMRU: Shoklo Malaria Research Unit
